# Non-Biased Enrichment Does Not Improve Quantitative Proteomic Delineation of Reovirus T3D-Infected HeLa Cell Protein Alterations

**DOI:** 10.3389/fmicb.2012.00310

**Published:** 2012-09-20

**Authors:** Jieyuan Jiang, Kolawole J. Opanubi, Kevin M. Coombs

**Affiliations:** ^1^Department of Medical Microbiology, Faculty of Medicine, University of ManitobaWinnipeg, MB, Canada; ^2^Manitoba Center for Proteomics and Systems Biology, University of ManitobaWinnipeg, MB, Canada; ^3^Manitoba Institute of Child Health, University of ManitobaWinnipeg, MB, Canada

**Keywords:** RNA virus, virus infection, host cell alterations, mass spectrometry, liquid chromatography, bioinformatics

## Abstract

Mass spectrometry-based methods have allowed elucidation of alterations in complex proteomes, such as eukaryotic cells. Such studies have identified and measured relative abundances of thousands of host proteins after cells are infected with a virus. One of the potential limitations in such studies is that generally only the most abundant proteins are identified, leaving the deep richness of the cellular proteome largely unexplored. We differentially labeled HeLa cells with light and heavy stable isotopic forms of lysine and arginine and infected cells with reovirus strain T3D. Cells were harvested at 24 h post-infection. Heavy-labeled infected and light-labeled mock-infected cells were mixed together 1:1. Cells were then divided into cytosol and nuclear fractions and each fraction analyzed, both by standard 2D-HPLC/MS, and also after each fraction had been reacted with a random hexapeptide library (Proteominer^®^ beads) to attempt to enrich for low-abundance cellular proteins. A total of 2,736 proteins were identified by two or more peptides at >99% confidence, of which 66 were significantly up-regulated and 67 were significantly down-regulated. Up-regulated proteins included those involved in antimicrobial and antiviral responses, GTPase activity, nucleotide binding, interferon signaling, and enzymes associated with energy generation. Down-regulated proteins included those involved in cell and biological adhesion, regulation of cell proliferation, structural molecule activity, and numerous molecular binding activities. Comparisons of the *r*^2^ correlations, degree of dataset overlap, and numbers of peptides detected suggest that non-biased enrichment approaches may not provide additional data to allow deeper quantitative and comparative mining of complex proteomes.

## Introduction

The mammalian reoviruses (MRV) are non-enveloped viruses with genomes consisting of 10 segments of double-stranded RNA. MRV is the prototype member of the *Orthoreovirus* genus in the Reoviridae family and was first isolated in the respiratory and enteric tracts of healthy humans in the early 1950s. MRV infections are generally mild in humans. The Orthoreoviruses include non-fusogenic MRV and fusogenic avian reovirus. MRV consist of three serotypes. Each serotype has prototype strains: strain Lang (T1L) for serotype 1, strain Jones (T2J) for serotype 2, and strain Dearing (T3D) for serotype 3 (Tran and Coombs, 2006; Schiff et al., 2007). One of the most potentially useful characteristics of MRV is its ability to selectively kill certain cancer cells (Coffey et al., 1998; Forsyth et al., 2008; Thirukkumaran et al., 2010). An activated Ras pathway and functional p53 appear to be requirements for this selective oncolytic property (Coffey et al., 1998; Pan et al., 2011). Global analyses of oligonucleotide microarrays have detected activation of numerous cellular genes, including many related to apoptosis (Poggioli et al., 2002; DeBiasi et al., 2003). However, global alterations in proteins (the effector molecules) after MRV infection have not yet been reported.

Except for certain epigenetic events (reviewed in Goldberg et al., 2007), a cell’s genome generally remains relatively constant. However, the cell’s proteome (the total protein repertoire, including all co-translational and post-translational modifications) varies greatly due to its biochemical interactions with the genome, as well as the cell’s interactions with the environment. In the case of viruses, which require the host cell’s machinery and metabolism to replicate, the cell’s proteome also reflects the specific alterations of the pathways induced by virus infection.

Previous analyses of how cells respond to virus infection have used microarray technologies which measure the cellular “transcriptome” (see for example; Geiss et al., 2002; Kobasa et al., 2007). However, there frequently is little concordance between microarray and protein data (Tian et al., 2004; Baas et al., 2006), partly because mRNA levels cannot provide complete information about levels of protein synthesis or extents of post-translational modifications. Thus, proteomic analyses have also been employed to better understand host alterations induced by virus infection. These have included two-dimensional difference in gel electrophoresis (2D-DIGE; see for examples; Burgener et al., 2008; Lucitt et al., 2008), isotope coded affinity tags (ICAT; Booy et al., 2005; Stewart et al., 2006), isobaric tags for relative and absolute quantitation (iTRAQ; Dwivedi et al., 2009; Zhang et al., 2009), and stable isotope labeling by amino acids in cell culture (SILAC; Skiba et al., 2008). We have previously used SILAC to measure proteomic alterations in influenza virus-infected A549 cells (Coombs et al., 2010). Cells were labeled with either ^12^C_6_-Lys and^12^C_6_^14^N_4_-Arg (“light”; **L**), or^13^C_6_-Lys and ^13^C_6_^15^N_4_-Arg (“heavy”; **H**), because virtually every tryptic peptide is expected to contain an **L** or **H** label, thereby providing increased protein coverage. In addition, **L** and **H** samples are mixed together early in this process, thereby reducing sample-to-sample variability.

Most quantitative proteomic analyses succeed in identifying and measuring several 1,000 proteins. Head-to-head comparisons suggest SILAC identifies more proteins than other methods (reviewed in Coombs, 2011); however, the 3,000–5,000 identified in many such studies still represents a small fraction of the estimated entire eukaryotic proteome. It is generally assumed that high-abundance proteins are most easily detected and low-abundance proteins masked by other components (Zolotarjova et al., 2008). Some studies have attempted to deplete high-abundance proteins (for example Dwivedi et al., 2009) or to use methods to enrich for selected proteins (Jiang et al., 2007). Both of these methods potentially suffer from selective bias for specific proteins. We decided to attempt to enrich for low-abundance proteins by using Proteominer™ (PM) beads (Bio-rad), which consist of a “library” of 64 million random hexapeptides to non-selectively bind interacting partners. We succeeded in the current study in identifying and measuring 2,736 host proteins. Sixty six proteins were significantly up-regulated, including those involved in antimicrobial and antiviral responses, GTPase activity, nucleotide binding, interferon signaling, and enzymes associated with energy generation. Sixty seven proteins, including those involved in cell and biological adhesion, regulation of cell proliferation, structural molecule activity, and numerous molecular binding activities were significantly down-regulated. However, comparison of the numbers of proteins identified with or without PM enrichment suggests this type of non-biased enrichment may not contribute substantially to deeper proteomic elucidation.

## Materials and Methods

### Cells and viruses

#### Cell lines

Spinner-adapted mouse fibroblast L929 cells (L929) were grown in Joklik’s modified minimal essential medium (J-MEM; Gibco, Grand Island, NY, USA) supplemented with 6% fetal bovine serum (FBS; Hyclone, Rockford, IL, USA), and 2 mM l-glutamine as described (Berard and Coombs, 2009). *Reovirus* was grown according to standard lab practice (Berard and Coombs, 2009).

Human HeLa cells were routinely cultured in Dulbecco’s modified MEM (DMEM) supplemented with non-essential amino acids, sodium pyruvate, 0.2% (w/v) glucose, 10% FBS (Hyclone), and 2 mM l-glutamine. Cells were maintained as monolayers in 5% CO_2_ and were passaged by trypsinization 2–3 times each week. For SILAC labeling, cells were grown in DMEM media provided with a SILAC™ Phosphoprotein Identification and Quantification Kit (Invitrogen Canada Inc., Burlington, ON, Canada), supplemented as above (except without non-essential amino acids), and with 10% dialyzed FBS (Invitrogen Canada Inc.), plus 100 mg each of “light” (**L**) or “heavy” (**H**) l-lysine and l-arginine per liter of DMEM.

#### Viruses

*Reovirus* strain Type 3 Dearing (T3D) is a laboratory stock. Virus amplifications were routinely performed in L929 cell monolayers grown in the presence of 5% CO_2_ at 37°C, supplemented with J-MEM as described above, except with 3% FBS instead of 6% FBS in the cell culture media, 100 U/ml of penicillin, 100 μg/ml streptomycin sulfate, and 100 μg/ml amphotericin-B as previously described (Berard and Coombs, 2009).

#### Virus purification

Large amounts of reovirus T3D were grown in 1 l suspension L929 cell cultures and purified by routine procedures involving Vertrel-XF™ extraction and cesium chloride (CsCl) ultracentrifugation (Mendez et al., 2000). Purified virions were then dialyzed against D-Buffer (150 mM NaCl, 15 mM MgCl_2_, 10 mM Tris, pH 7.4). Virus concentration was measured by optical density at 260 nm, using the relationship 1 ODU = 2.1 × 10^12^ particles per milliliter (Smith et al., 1969) and infectivity was titrated.

#### Virus titrations

Serial 1:10 dilutions of virus samples were made in gel saline (137 mM NaCl, 0.2 mM CaCl_2_, 0.8 mM MgCl_2_, 19 mM HBO_3_, 0.1 mM Na_2_B_4_O_7_, and 0.3% w/v gelatin). HeLa cell and L929 cell monolayers in six-well plates were infected in duplicate, viruses allowed to attach to cells for 1 h with periodic rocking, and each well overlaid with a 50:50 ratio of 2% agar and 2× Medium 199 (M199) supplemented with a final concentration of 3% FBS, 2 mM l-glutamine, 100 U/ml of penicillin, 100 μg/ml streptomycin sulfate, and 100 μg/ml amphotericin-B. Plates were fed 3 days later with fresh agar/M199 and were stained with a 0.04% neutral red solution on day 6. Viral plaques were counted 15–18 h later and titers calculated (Berard and Coombs, 2009).

#### SILAC infection

Once HeLa cells had grown through six doublings in appropriate SILAC media, **H** cells were infected with gradient-purified T3D at a multiplicity of infection (MOI) of seven plaque forming units (PFU) per cell. An equivalent number of **L** cells were mock-infected with diluent as control. Cells were overlaid with appropriate media and cultured for 24 h.

### Cell fractionation

At 24hpi, **L** and **H** cells in the T75 flasks were collected and counted. To verify infection status of each culture, aliquots of all cultures were saved for virus titration. For comparative SILAC assays, equivalent numbers of **L** and **H** cells were mixed together, and the mixed cells were washed 3× in >50 volumes of ice-cold Phosphate Buffered Saline (PBS). Washed cells were lysed with 0.5% NP-40, supplemented with 1.1 μM pepstatin A, incubated on ice for 30 min, and nuclei removed by pelleting at 5,000 × *g* for 10 min. The cytosol and soluble membranes (supernatant) were transferred to a fresh microfuge tube; and the two fractions (nuclear pellet and supernatant) were frozen at −80°C until further processing took place.

Thawed nuclei were extracted with one volume of High Salt Buffer (620 mM NaCl, 1 mM DTT, 10 mM Tris, pH 8.0), insoluble material pelleted at 15,000 × *g* for 10 min, and the supernatant removed and saved. Insoluble pellets were then extracted with 1/3rd volume of 8 M urea, insoluble material pelleted as above, the two extractions combined, and samples stored at −80°C until further processing took place.

### Proteominer™ purification

Approximately 90% of each fraction (cytosol and nucleus) was passed through separate PM Mini columns. The columns were processed according to manufacturer’s protocol (Bio-Rad Corp). Briefly, the cytosolic and nuclear protein fractions were measured and each fraction concentrated to ≈20 mg/ml (∼1 ml). PM beads were washed twice with Wash Buffer then incubated with each concentrated protein sample for 2 h with end-to-end shaking. Columns were spun at 1,000 × *g* for 2 min to remove excess fluid, washed 3× with Wash Buffer, and then bound proteins eluted with two sequential applications of 200 μl One-step Elution Buffer.

### Western blotting

Western blot analyses of HeLa cells were performed essentially as described previously (Coombs et al., 2010). Briefly, unlabelled cells were harvested essentially as described above and cytosolic proteins were resolved on a 10% SDS-PAGE gel at 120 V for 70 min. Proteins were transferred to polyvinylidenedifluoride (PVDF) membranes at 20 V for 30 min in a semi-dry apparatus, and the transfer confirmed by Ponceau staining. Membranes were blocked with 5% skim milk in TBST and probed with various antibodies in 1% BSA in TBST. Primary antibodies were: in-house rabbit anti-reovirus, α-GAPDH (Cell Signaling, cat#2118), α-IFIT2 (Abcam, cat#ab55837), and α-SAMD9 (Sigma cat#HPA021318), goat α-Mx1 (Santa Cruz cat#sc-34128), and mouse anti-STAT1 (Cell Signaling, cat#9176), α-Actin (Sigma, cat#A5441). The secondary antibodies were the appropriate horseradish peroxidase (HRP)-conjugated rabbit anti-mouse or goat anti-rabbit (Cell Signaling, cat#7076 and cat#7074, respectively). Bands were detected by enhanced chemiluminescence using an Alpha Innotech FluorChem Q Multi Image III instrument.

### Immunofluorescent microscopy

HeLa cells were grown overnight in a 37°C, 5% CO_2_ incubator to 80% confluency on autoclaved 12-spot slides and then infected with MRV T3D at a MOI of seven or mock-infected. Mock, 0, 6, 12, and 24 h infected cells were washed 5× with PBS and fixed with 4% paraformaldehyde for 15 min at 4°C. Cells were then washed 4× with 1× PBS and kept in 1× PBS at 4°C until the 24 h time point was collected. Cells from all time points were then permeabilized with 0.1% TritonX-100 in 1× PBS for 5 min at 4°C followed by five washes with 1× PBS. Cells were blocked with 1% BSA in 1× PBS and then treated with primary antibody (in-house rabbit anti-reovirus). Cells were then washed 5× with 1× PBS and treated with Alexa Fluor^®^ 488 Goat anti-Rabbit (Invitrogen, cat#A11008) secondary antibody (all antibodies were diluted in 1% BSA in 1× PBS). Cells were then washed 5× with 1× PBS and Anti-fade prolong gold reagent with DAPI (Invitrogen, Cat# P36935) was added to each spot before slides were covered with coverslips, dried, and sealed. Slides were examined on a Zeiss Axio Observer Z1 inverted microscope using 10 and 20× objectives and fluorescence illumination using ExfoXcite. Images were acquired using AxioVision 4.8.2 software.

### Protein digestion

Protein content in the non-purified (“standard”) and PM-purified cytosolic and nuclear fractions collected as described above were determined using a BCA™ Protein Assay Kit (Pierce; Rockford, IL, USA) and BSA standards. After protein concentration determinations, samples were diluted with freshly made 100 mM ammonium bicarbonate to provide concentrations of ∼1 mg/ml and pH ∼ 8. Three hundred microliters of each sample (∼300 μg of protein) were reduced, alkylated, and trypsin digested as previously described (Coombs et al., 2010). Briefly, 30 μl of freshly prepared 100 mM dithiothreitol (DTT) in 100 mM ammonium bicarbonate was added, incubated for 45 min at 60°C, 30 μl of freshly prepared iodoacetic acid (500 mM solution in 100 mM ammonium bicarbonate) was added, and the tubes were then incubated for 30 min at room temperature, in the dark. Finally, 50 μl of 100 mM DTT solution was added to quench the excess iodoacetic acid. Samples were digested overnight at 37°C with 6 μg of sequencing grade trypsin (Promega, Madison, WI, USA). The samples were lyophilized and stored at −80°C.

### Peptide fractionation using 2D RP HPLC

A newly developed orthogonal procedure (Gilar et al., 2005; Spicer et al., 2007) was employed for 2D RP (reversed-phase) high pH – RP low pH peptide fractionation. Lyophilized tryptic digests were dissolved in 200 μl of 20 mM ammonium formate pH 10 (buffer A for first dimension separation), injected onto a 1 mm × 100 mm XTerra (Waters, Milford, MA, USA) column and fractionated using a 0.67% acetonitrile per minute linear gradient (Agilent 1100 Series HPLC system, Agilent Technologies, Wilmington, DE, USA) at a 150 μl/min flow rate. Sixty one-minute fractions were collected (covering ∼40% acetonitrile concentration range) and concatenated using procedures described elsewhere (Spicer et al., 2007; Dwivedi et al., 2008); the last 30 fractions were combined with the first 30 fractions in sequential order (i.e., #1 with #31; #2 with #32, etc.). Combined fractions were vacuum-dried and re-dissolved in buffer A for the second dimension RP separation (0.1% formic acid in water).

A split less nano-flow Tempo LC system (Eksigent, Dublin, CA, USA) with 20 μl sample injection via a 300 μm × 5 mm PepMap 100 pre-column (Dionex, Sunnyvale, CA, USA) and a 100 μm × 200 mm analytical column packed with 5 μm Luna C18(2; Phenomenex, Torrance, CA, USA) were used in the second dimension separation prior to MS analysis. Both eluents A (water) and B (acetonitrile) contained 0.1% formic acid as an ion-pairing modifier. A 0.33% acetonitrile per minute linear gradient (0–30% B) was used for peptide elution, providing a total 2 h run time per fraction in the second dimension.

### Mass spectrometry, bioinformatics, and data mining

A QStar Elite mass spectrometer (Applied Biosystems, Foster City, CA, USA) was used in a data-dependent MS/MS acquisition mode. One-second survey MS spectra were collected (*m*/*z* 400–1,500) followed by MS/MS measurements on the three most intense parent ions (80 counts/s threshold, +2 ±4 charge state, *m*/*z* 100–1,500 mass range for MS/MS), using the manufacturer’s “smart exit” (spectral quality five) settings. Previously targeted parent ions were excluded from repetitive MS/MS acquisition for 60 s (50 mDa mass tolerance). Raw data files (30 in total for each run) were submitted for simultaneous search using standard SILAC settings for QStar instruments and were analyzed by Protein Pilot^®^, version 4.0, using the non-redundant human gene database. A decoy database search strategy (NCBInr *Homo sapiens* in which all protein sequences were reversed) was used to estimate the false discovery rate, which for this dataset was <0.8%. Proteins, and their confidences and **H:L** ratios, were returned with GeneInfo Identifier gi accession numbers. Proteins for which at least two fully trypsin digested **L** and **H** peptides were detected at >99% confidence were used for subsequent comparative quantitative analysis.

Differential regulation within each experimental dataset was determined by normalization of each dataset, essentially as described (Keshamouni et al., 2009). Briefly, every **H:L** ratio was converted into log_2_ space to determine geometric means and facilitate normalization. The average log_2_ **H:L** ratios and SDs of the log_2_ **H:L** ratios were determined for each dataset. Every proteins’ log_2_ **H:L** ratio was then converted into a *z*-score, using the formula:

$$\text{Z - score (}\sigma \text{) of [}b\text{]=}\frac{\begin{array}{c} {\text{Log}}_{\text{2}}\text{H:L [}b\text{] – average of}\\ {\text{(log}}_{\text{2}}\text{ of each member, }a\;\ldots \;n\text{)} \end{array}}{\begin{array}{c} \text{standard deviation of}\\ {\text{(log}}_{\text{2}}\text{ of each member, }a\;\ldots \;n\text{)} \end{array}}$$

where “*b*” represents an individual protein in a dataset population *a*…*n*, and *z*-score is the measure of how many SD units (expressed as “σ”) that protein’s log_2_ **H:L** ratio is away from its population mean. Thus, a protein with a *z*-score >1.645σ indicates that protein’s differential expression lies outside the 90% confidence level, >1.960σ indicates outside the 95% confidence level, 2.576σ indicates 99% confidence, and 3.291σ indicates 99.9% confidence. *z*-Scores >1.960 were considered significant. gi numbers of all significantly regulated proteins were converted into HGNC identifiers by Uniprot^1^ and HGNC terms were submitted to and analyzed by the DAVID bioinformatic suite at the NIAID, version 6.7 (Dennis et al., 2003; Huang et al., 2009a) and gene ontologies examined with the “FAT” datasets. The gi numbers were also submitted to, and pathways constructed with, Ingenuity Pathway Analysis software (IPA^®^).

## Results and Discussion

### Identification of altered host proteins

**Figure 1:**
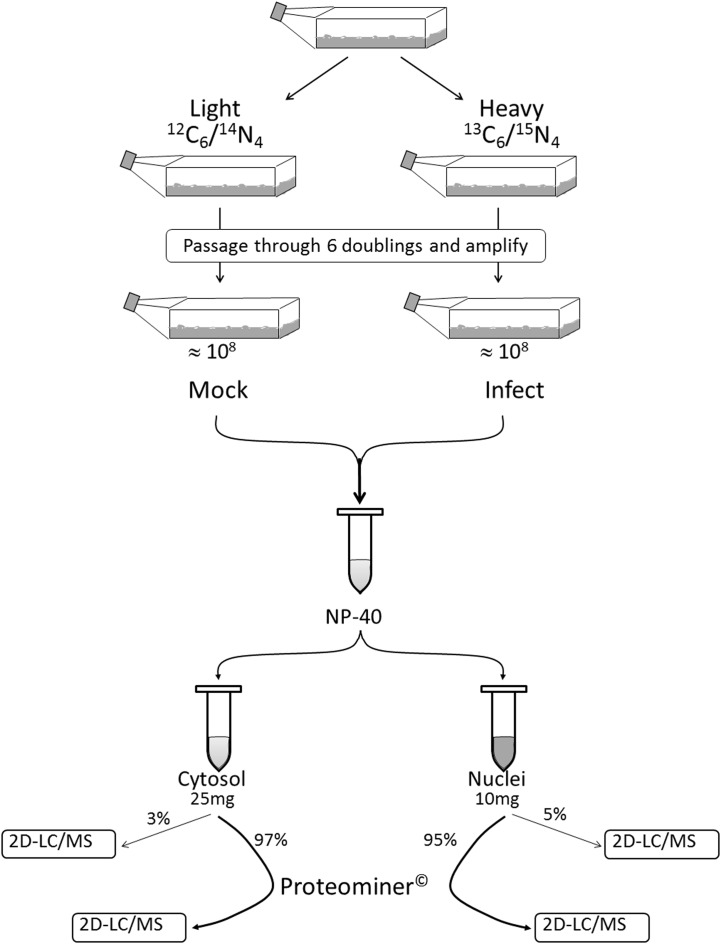
**Outline of experimental set-up**. Cells were passaged through six doublings in either **L**ight or **H**eavy SILAC medium and the **H** cells infected with reovirus T3D. Infected (**H**) and mock-infected (**L**) cells were mixed together 1:1. After the cells were washed and lysed to separate cytosol from nucleus, 95–95% of each fraction was non-specifically enriched for low-abundance proteins by reaction with Proteominer^™^(PM) beads. Each of the four fractions (two PM-enriched as well as two residual 3–5% “standard” fractions) were then processed by 2D-HPLC/MS.

**Figure 2:**
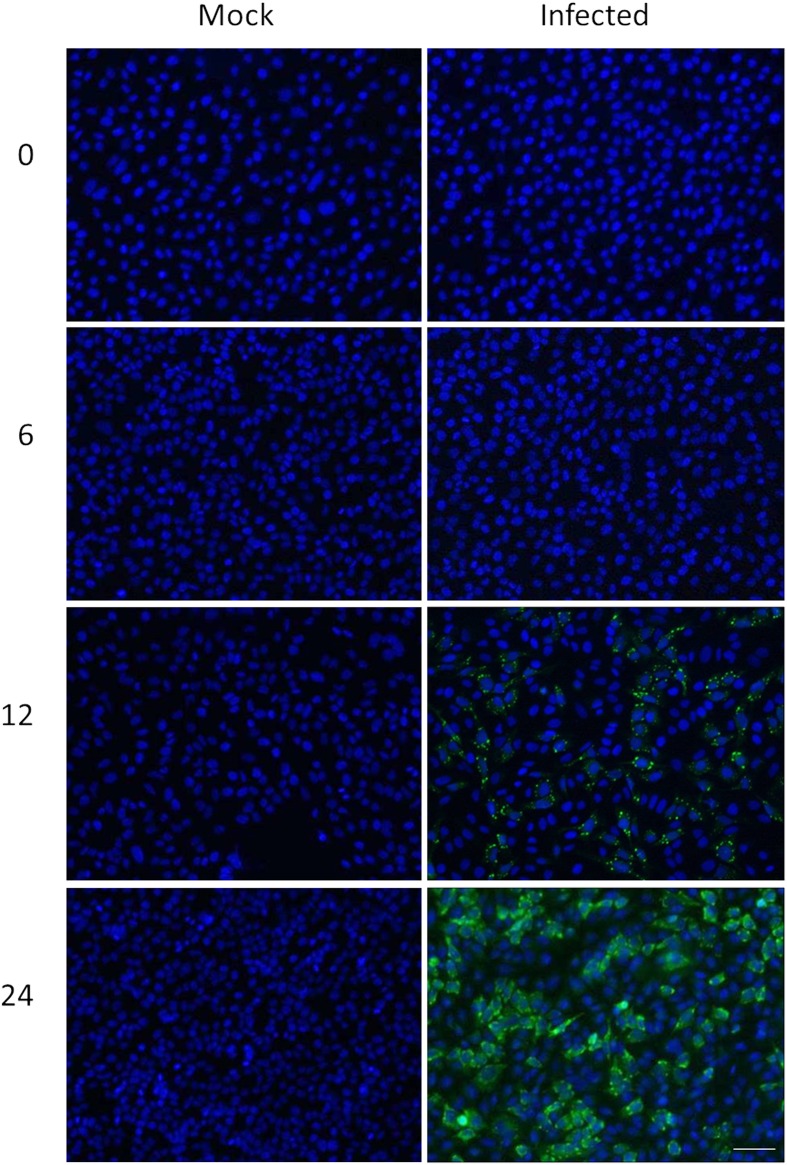
**Confirmation of HeLa cell infectivity**. HeLa cells were mock-infected (left), or infected with MRV strain T3D at an MOI of 7 (right). Cells were harvested at indicated times post-infection (left) and processed for immunofluorescence microscopy, using in-house rabbit anti-reovirus and Alexa-488-conjugated secondary anti-rabbit antibody (green) and DAPI (blue). Scale bar is 50 μm.

**Figure 3:**
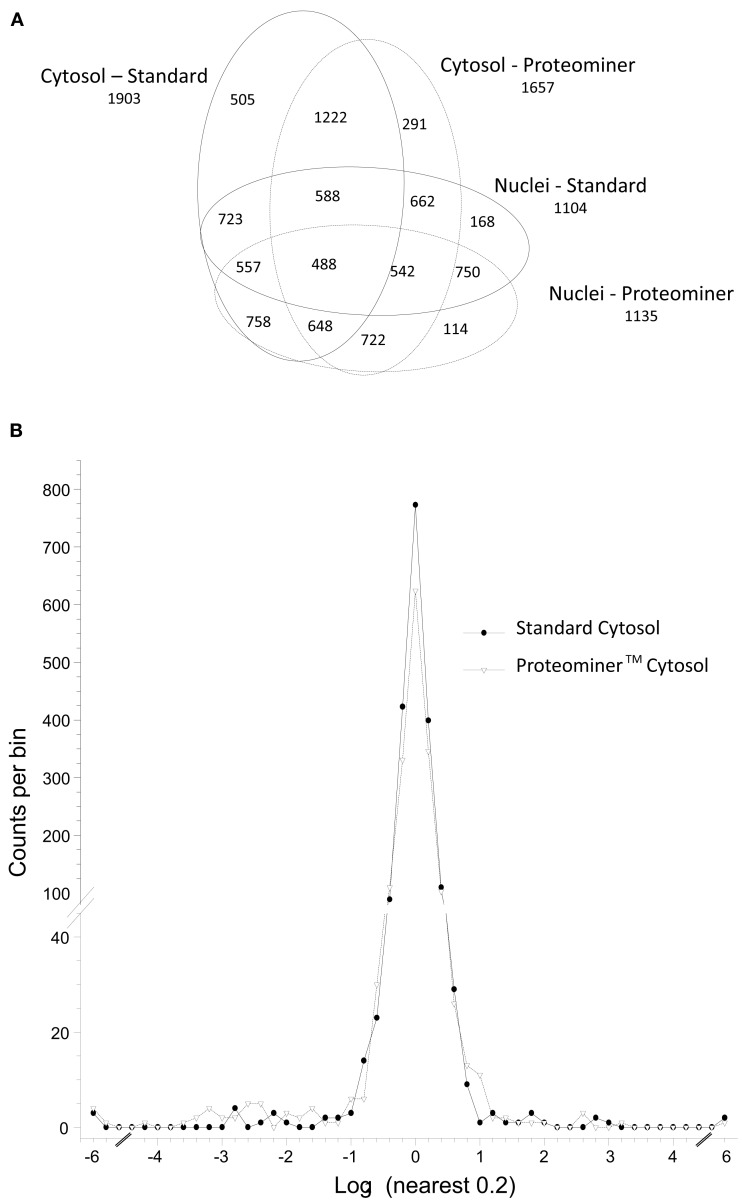
**Distributions of proteins identified in various experiments**. **(A)** Venn diagram of numbers of identified proteins from various analyses. **(B)** Frequency distributions of identified proteins in two virus-infected sample sets, with **H:L** ratios expressed as log_2_ values. Positive values represent up-regulated host proteins in virus-infected cells; negative values represent down-regulated host proteins. Characteristics of all peptide and protein distributions, mean log_2_ **H:L** ratios, and SDs of log_2_ **H:L** ratios are shown in Table 1.

We combined ∼10^8^ **H**-labeled reovirus-infected HeLa cells with an equivalent amount of **L**-labeled non-infected cells, lysed the cells to generate cytosolic and nuclear fractions, and reacted ∼95% of each fraction with a commercially available random hexapeptide library (PM™) to enrich for low-abundance proteins. This strategy was chosen to attempt to complement the proteomic coverage of high-abundance and medium-abundance proteins expected from standard 2D-HPLC/MS processing (outlined in Figure 1). We also confirmed that the majority of HeLa cells demonstrated virus replication under our experimental conditions by 12–24hpi, as measured by immunofluorescent microscopy (Figure 2). Our standard 2D-HPLC/MS process identified 2,472 proteins from 21,989 non-redundant **H:L** peptide pairs in the cytosolic fraction. However, exclusion of those proteins whose identification confidence was <99% reduced the number of identified proteins to 1,903 (Table 1; Figure 3A). Using similar criteria, we found 1,657 proteins at ≥99% confidence in the cytosolic fraction reacted with the PM library and about 1,100 proteins in each of the nuclear fractions. Since crude nuclear fractions were frozen and no attempts were made to remove traces of cytosolic proteins from this fraction, these assays were meant to provide additional cell fractions rather than to allow meaningful distributional characterization and the “nuclear” fractions were expected to be contaminated with some cytosolic proteins.

**Table 1:** **Number of peptides, proteins, log_2_**H:L** ratio means and SD, and *z*-scores of SILAC-measured HeLa cell proteins**.

	Cytosol		Nuclei	
	Standard^1^	Proteominer^2^	Standard	Proteominer
Total number of peptide pairs^3^	24,927	17,484	14,594	13,108
Total number of proteins^4^	1,903	1,657	1,104	1,135
Number of proteins analyzed^5^	1,838	1,570	1,047	1,064
Mean log_2_ H:L ratios	0.0124	0.0009	0.0055	0.0156
SD of log_2_ H:L ratios	0.2759	0.3526	0.3035	0.3314
Number of proteins at *z*-score cutoff of: ±1.960σ (95%)	40, 33^6^	34, 27	32, 29	18, 20
±2.576σ (99%)	21, 17	19, 20	14, 15	14, 15
±3.291σ (99.9%)	14, 5	8, 17	11, 7	8, 11

*^1^Indicated cellular fraction was trypsinized and directly processed by two-dimensional HPLC/MS*.

*^2^Indicated cellular fraction was incubated with Proteominer^™^ beads, eluted, trypsinized, and processed by 2-D HPLC/MS*.

*^3^Total number of **H:L** peptide pairs for all proteins identified at confidence level ≥99%*.

*^4^Total number of proteins identified at confidence level ≥99%*.

*^5^Number of proteins analyzed after those identified by only a single peptide, as well as possible contaminants, removed*.

*^6^First value is number of up-regulated proteins outside the indicated confidence level; second number is number of down-regulated proteins outside the indicated confidence level*.

Combination of all fractions, and removal of all proteins identified by only a single peptide, resulted in identification and measurement of 2,759 total unique protein pairs. Each protein’s **H:L** ratio was converted to log space and inspection of each dataset indicated variability in each dataset’s mean log_2_ value and in each dataset’s log_2_ SD (Figure 3B; Table 1). Thus, every proteins’ **H:L** ratio was converted into a *z*-score as described in Section “Materials and Methods” (and in Coombs et al., 2010) to facilitate comparisons of each dataset. A number of proteins with significantly high or low log_2_ values and corresponding *z*-scores represented keratins and other proteins identified in other studies as probable contaminants (i.e., S200 binding proteins); thus, these proteins were removed from further calculations.

Stratification of each protein’s **H:L** ratio and its corresponding *z*-score indicated that numerous proteins in each sample could be considered significantly regulated. For example, of the 1,838 proteins identified in the standard cytosolic preparation, 40 were up-regulated at 95% confidence and 14 were also up-regulated at 99.9% confidence (Table 1). Thirty three proteins in the same dataset were down-regulated at 95% confidence, and five of these proteins were also down-regulated at 99.9% confidence. Inspection of protein **H:L** ratios and *z*-scores indicated that most proteins differentially regulated at >95% confidence had **H:L** ratios altered by >1.5-fold. Thus, proteins observed more than a single time were considered significantly regulated if at least one of their observations had a *z*-score ≥1.960σ, if another observation in the same type of fraction (i.e., standard cytosolic and PM cytosolic) was no more than 0.75σ in the opposite direction, and if the average **H:L** ratio was >1.5-fold. Using the above criteria, we identified and measured 66 proteins that were significantly up-regulated and 67 proteins that were significantly down-regulated (Table 2).

**Table 2:** **Significantly affected HeLa cell proteins after reovirus infection**.

Accession	HGNC ID	Name	Cytoplasm					Nucleus				
				Standard		Proteominer			Standard		Proteominer	
			Inf/Mock^1^	# Peps	*z*-Score	#Peps	*z*-Score	Inf/Mock^1^	# Peps	*z*-Score	# Peps	*z*-Score
**UP-REGULATED PROTEINS**												
**Proteins detected in multiple similar fractions**												
gi|8923450	SDHAF2	Succinate dehydrogenase assembly factor 2, mitochondrial precursor	50.6	4	**24.036^1^**	4	0.526					
gi|222136619	MX1	Myxovirus resistance protein 1	6.12	6	**6.583**	6	**8.839**	42.0	2	**21.873**	3	**5.234**
gi|116534937	IFIT1	Interferon-induced protein with tetratricopeptide repeats 1 isoform 2	6.45	6	**9.877**	4	**7.414**	2.67			2	**4.227**
gi|55741675	K0907	Hypothetical protein LOC22889						4.59	3	−0.368	3	**9.139**
gi|4826649	RM49	Mitochondrial ribosomal protein L49	4.58	2	**10.901**	2	0.178	0.95			8	−0.266
gi|4826774	ISG15	ISG15 ubiquitin-like modifier	3.80	10	**7.170**	5	**5.083**	3.29			4	**5.131**
gi|27881482	DDX58	DEAD/H (Asp-Glu-Ala-Asp/His) box polypeptide RIG-I	3.77	2	**6.322**	2	**5.832**					
gi|6274552	STAT1	Signal transducer and activator of transcription 1 isoform alpha	2.76	19	**5.372**	8	**3.952**	1.81	3	−1.456	6	**3.663**
gi|72534658	IFIT3	Interferon-induced protein with tetratricopeptide repeats 3	2.57	2	0.803	5	4.660	1.02			1	
gi|19743875	FUMH	Fumaratehydratase precursor	1.02	25	0.240	16	−0.136	2.24	4	**5.851**	4	0.098
gi|4507241	SSRP1	Structure specific recognition protein 1	2.12	8	**2.640**	1		0.01	2	**−21.042**		
gi|4506103	E2AK2	Eukaryotic translation initiation factor 2-alpha kinase 2 isoform a	1.95	6	**2.760**	10	**3.010**	1.59			6	**1.972**
gi|4506003	PP1A	Protein phosphatase 1, catalytic subunit, alpha isoform 1	0.94	23	−0.352			1.91	3	**4.587**	3	0.699
gi|112789562	IF16	Interferon, gamma-inducible protein 16	1.70	7	**2.314**	3	**2.858**	1.90	7	**2.219**	4	**3.824**
gi|42516576	GLRX5	Glutaredoxin 5	1.84	3	1.658	4	3.180					
gi|166706903	GBP1	Guanylate binding protein 1, interferon-inducible, 67 kDa	1.81	10	**2.380**	9	**2.933**					
gi|38016914	SAMH1	SAM domain- and HD domain containing protein 1	1.71	2	**4.006**	4	1.599					
gi|50592994	THIO	Thioredoxin	0.98	8	−0.430	11	0.070	1.69	2	−0.620	6	**2.876**
gi|48762920	K6PL	Liver phosphofructokinase	1.69	5	0.603	3	3.920					
gi|52630342	1C07	Major histocompatibility complex, class I, C precursor	1.67	14	0.382	16	3.194	1.27			3	1.007
gi|22035653	APOL2	Apolipoprotein L2	1.64	3	**2.583**	2	1.951					
gi|5031777	IDH3A	Isocitrate dehydrogenase 3 NAD(+) alpha precursor	0.97	18	−0.385	15	0.062	1.57	2	**2.162**	2	1.892
gi|223718097	OXA1L	Oxidase (cytochrome *c*) assembly 1-like	1.57	1		2	2.576					
gi|19923973	KCD12	Potassium channel tetramerization domain containing 12	1.07	7	0.328			1.57	2	**1.969**	3	**1.999**
gi|4758786	NDUS2	NADH dehydrogenase (ubiquinone) Fe-S protein 2	1.55	3	**2.985**	2	0.692	1.56			3	1.880
gi|5031863	LG3BP	Galectin 3 binding protein	1.55	7	1.474	13	**2.083**					
gi|62530384	ECI1	dodecenoyl-Coenzyme A delta isomerase precursor	0.99	12	−0.313	2	0.730	1.55	4	**2.281**	3	1.564
gi|9506689	EXOS4	Exosome component 4	1.08			5	0.297	1.53	2	**4.810**	5	0.132
**Proteins detected multiple times/regulated at least once**		
gi|33356547	MCM2	Minichromosome maintenance complex component 2	1.13	28	0.439	28	0.584	8.04			3	**9.026**
gi|5453740	ML12A	Myosin, light chain 12A, regulatory, non-sarcomeric	6.89	13	**10.050**			0.84			10	−0.811
gi|24307901	IFI35	Interferon-induced protein 35	3.71	2	**6.811**			3.04	3	**5.269**		
gi|5174513	SMAD3	mothers against decapentaplegic homolog 3 isoform 1	1.23			1		2.03			3	**3.044**
gi|4503049	CRIP2	Cysteine-rich protein 2	1.99			4	**2.809**	1.18			3	0.677
gi|148747351	PACN2	Protein kinase C and casein kinase substrate in neurons 2	1.70	3	**2.742**			1.04			2	0.103
gi|21956645	MTPN	Myotrophin	1.07	3	0.323			1.68			2	**2.211**
gi|33469966	SCFD1	Vesicle transport-related protein isoform a	0.98	3	1.814	8	−0.875	1.68	4	**2.439**		
gi|5902076	SRSF1	Splicing factor, arginine/serine-rich 1 isoform 1	1.63			2	**1.994**	1.07	14	0.109	25	0.336
gi|39780588	TSR1	TSR1, 20S rRNA accumulation	1.00	1				1.58	4	**2.147**		
gi|13540606	CLPB	Caseinolytic peptidase B	1.52	2	**2.158**			0.49	1			
**Proteins detected once**		
gi|17921993	TBA3C	Tubulin, alpha 3c	100	86	**24.036**							
gi|31543983	ARFG2	ADP-ribosylation factor GTPase activating protein 2						5.73	3	**8.278**		
gi|4758442	GMFB	Glia maturation factor, beta	3.03	2	**5.754**							
gi|19923597	SP130	Sin3A-associated protein, 130 kDa isoform b						2.49	2	**4.318**		
gi|13375616	FADS3	Fatty acid desaturase 3	2.14	2	**3.931**							
gi|74271837	GLNA	Glutamine synthetase	2.01			3	**2.852**					
gi|4502209	ARF5	ADP-ribosylation factor 5						1.90			3	**2.747**
gi|70608211	NT5C3	5(-Nucleotidase, cytosolic III isoform 2	1.88			3	**2.587**					
gi|20631967	BAX	Apoptosis regulator BAX isoform sigma	1.87			2	**2.559**					
gi|4757876	BST2	Bone marrow stromal cell antigen 2						1.87			4	**2.666**
gi|222144328	MYL12B^3^	Myosin regulatory light chain MRCL2 isoform B						1.83	6	**2.847**		
gi|53828918	PGTA	Rabgeranylgeranyltransferase alpha	1.83			2	**2.459**					
gi|190014625	RRP44	DIS3 mitotic control isoform b						1.81	2	**2.810**		
gi|5729820	SYFM	Phenylalanyl-tRNAsynthetase 2 precursor	1.79			2	**2.382**					
gi|4505467	NT5E	5′ Nucleotidase isoform 1 preproprotein						1.74	2	**2.618**		
gi|4505895	PLRG1	Pleiotropic regulator 1 (PRL1 homolog, *Arabidopsis*)						1.71	2	**2.540**		
gi|4505587	PA1B3	Platelet-activating factor acetylhydrolase, isoform Ib, gamma subunit	1.67	2	**2.634**							
gi|28395033	RHOC	Ras homolog gene family, member C precursor	1.62			18	**1.981**					
gi|148536825	CO4A1	Alpha 1 type IV collagen preproprotein	1.59	2	**2.380**							
gi|71044479	DIDO1	Death inducer-obliterator 1 isoform c						1.58	3	**2.153**		
gi|9955963	ABCB6	ATP-binding cassette, sub-family B, member 6	1.56	4	**2.294**							
gi|56676335	RIF1	RAP1 interacting factor 1						1.56	2	**2.083**		
gi|40254978	FIP1	FIP1 like 1 isoform 1						1.54	4	**2.041**		
gi|7706481	CAB39	Calcium binding protein 39	1.54	5	**2.203**							
gi|221316634	LMO7	LIM domain only 7 isoform 2						1.53	6	**1.991**		
gi|194473714	LXN	Latexin	1.52	3	**2.155**							
gi|8923219	TRM1	tRNAmethyltransferase 1 isoform 1	1.50	6	**2.072**							
**DOWN-REGULATED PROTEINS**												
**Proteins detected in multiple similar fractions**												
gi|4507241	SSRP1	FACT complex subunit SSRP1	2.12	8	**2.640**	1		0.012	2	**−21.042**		
gi|4506457	RCN2	Reticulocalbin 2 precursor	0.86	14	−0.076	27	−0.993	0.32			2	**−4.994**
gi|4505751	PROF2	Profilin 2 isoform b	0.91	8	−0.215	9	−0.601	0.46			3	**−3.447**
gi|7661832	SSU72	Ssu72 RNA polymerase II CTD phosphatase homolog	0.84	3	−1.873	3	−0.127	0.52			3	**−2.902**
gi|4506929	SH3G1	SH3 domain GRB2-like 1	0.57	5	**−3.424**	3	−1.835	27.85	3	**21.873**	8	−1.062
gi|72534660	SRSF7	Splicing factor, arginine/serine-rich 7	0.57	2	0.155	5	**−3.972**	0.91	6	−1.680	12	0.031
gi|7661672	PDIP2	DNA polymerase delta interacting protein 2	0.88	5	−0.696	3	−0.516	0.58			2	**−2.426**
gi|4758340	SYFA	Phenylalanyl-tRNAsynthetase, alpha subunit	0.96	9	−0.532	13	−0.003	0.58	4	**−2.095**	6	**−2.721**
gi|31543415	G45IP	Growth arrest and DNA-damage-inducible, gamma interacting protein 1						0.60	3	**−21.042**	4	0.149
gi|45359846	G3BP2	Ras-GTPase activating protein SH3 domain-binding protein 2 isoform b						0.60	12	**−3.099**	9	−1.520
gi|4507467	BGH3	Transforming growth factor, beta-induced, 68kDa precursor	0.64	7	**−3.152**	6	−1.196	0.82			5	−0.890
gi|40353740	LARP4	La-related protein 4 isoform b	0.65	1		3	**−2.585**	1.27			2	0.997
gi|4503523	EIF3D	Eukaryotic translation initiation factor 3 subunit D	1.07	8	0.672	3	−0.582	0.66	4	**−2.237**	1	
**Proteins detected multiple times/regulated at least once**		
gi|16554629	WDR5	WD repeat domain 5	0.011	4	**−23.627**			1.00	2	0.001	2	−0.034
gi|4502337	ZA2G	Alpha-2-glycoprotein 1, zinc	0.11			2	**−8.924**	0.012			2	**−19.301**
gi|60097902	FLG	Filaggrin	0.01			2	**−18.099**	1.99			1	
gi|4557894	LYSC	Lysozyme precursor	0.11			5	**−8.960**	0.17			1	
gi|4505821	PIP	Prolactin-induced protein	0.33			3	**−4.601**	0.29			2	−5.451
gi|58530840	DESP	Desmoplakin isoform I	0.30			11	**−4.902**	0.47			2	−3.371
gi|8922652	ARFG1	ADP-ribosylation factor GTPase activating protein 1 isoform a	1.07			4	0.290	0.46			2	−3.437
gi|116235460	YTHD3	YTH domain family, member 3	0.50			4	**−2.822**	0.88	5	**−3.099**	11	0.145
gi|13129040	SPATA5L1	Spermatogenesis associated 5-like 1	1.12			2	0.454	0.52	2	**−3.090**		
gi|145580575	CTBP2	C-Terminal binding protein 2 isoform 2	1.07			9	0.282	0.53			4	**−2.827**
gi|4826730	MTOR	FK506 binding protein 12-rapamycin associated protein 1	0.99	2	−0.092			0.55	4	**−2.860**		
gi|47271443	SRSF2	Splicing factor, arginine/serine-rich 2	0.56	4	**−3.068**			1.35	5	−1.187	3	**3.590**
gi|4506901	SRSF3	Splicing factor, arginine/serine-rich 3	0.60			5	**−2.120**	0.99	12	0.465	17	−0.448
gi|4885245	FOSL2	FOS-like antigen 2	0.91			2	−0.402	0.60			2	**−2.271**
gi|20127486	PLIN3	Perilipin-3 isoform 1	1.00	24	−0.045			0.61	3	**−2.391**		
gi|7657176	CNPY2	Canopy 2 homolog	0.96	12	−0.242			0.61	2	**−2.337**		
gi|56118310	NUCKS	Nuclear casein kinase and cyclin-dependent kinase substrate 1	1.01	4	0.017			0.66	6	**−2.022**		
gi|89276751	CO5A1	Alpha 1 type V collagen preproprotein	0.86	10	−0.815			0.66	3	**−2.022**		
**Proteins detected once**		
gi|4885477	MYG	Myoglobin	0.012			2	**−18.099**					
gi|61835172	FXR1	Fragile X mental retardation-related protein 1 isoform c						0.012			2	**−19.301**
gi|119703744	DSG1	Desmoglein 1 preproprotein	0.10			4	**−9.424**					
gi|62122917	FILA2	Filaggrin family member 2	0.11			3	**−9.185**					
gi|54607120	TRFL	Lactotransferrin precursor	0.12			5	**−8.747**					
gi|189458821	TGM3	Transglutaminase 3 precursor	0.18			2	**−6.951**					
gi|38348366	SBSN	Suprabasin						0.21			2	**−6.925**
gi|4885165	CYTA	Cystatin A						0.22			2	**−6.580**
gi|15187164	LACRT	Lacritin precursor	0.24			2	**−5.876**					
gi|170296790	A8CED1	Mesotrypsin isoform 1 preproprotein						0.25	6	**−6.665**		
gi|239755818	LOC100293351^3^	PREDICTED: hypothetical protein isoform 2	0.35			2	**−4.275**					
gi|221316620	CD123	Cell division cycle 12	0.38	3	**−5.063**							
gi|116686122	KIF4A	Kinesin family member 4						0.42	2	**−4.187**		
gi|4501889	ACTH	Actin, gamma 2 propeptide	0.44	85	**−4.302**							
gi|14327896	CCNB1	cyclin B1						0.48	2	**−3.487**		
gi|48762942	HIP1R	Huntingtin interacting protein-1-related						0.50	2	**−3.304**		
gi|155722990	SLC4A1AP	Kanadaptin						0.53	2	**−3.018**		
gi|50658084	BCAT2	Branched chain aminotransferase 2, mitochondrial	0.55	4	**−3.200**							
gi|47825361	NCRP1	Non-specific cytotoxic cell receptor protein 1 homolog	0.56			2	**−2.346**					
gi|4502951	CO3A1	Collagen type III alpha 1 preproprotein	0.57	5	**−2.948**							
gi|114796644	RCC1	Regulator of chromosome condensation 1 isoform a	0.58	3	**−2.920**							
gi|7705999	TMEM9	Transmembrane protein 9	0.58	2	**−2.911**							
gi|19882251	CYTN	Cystatin SN precursor	0.58			3	**−2.238**					
gi|154240704	TM192	Transmembrane protein 192	0.58			2	**−2.210**					
gi|82546824	FOXK1	Forkhead box K1	0.58	3	**−2.866**							
gi|190684694	UBP8	Ubiquitin specific peptidase 8						0.59	2	**−2.567**		
gi|221219053	DNAJC7	DnaJ (Hsp40) homolog, sub-family C, member 7 isoform 1	0.59	3	**−2.795**							
gi|7657655	TRAM1	Translocation associated membrane protein 1						0.63	3	**−2.199**		
gi|4557555	EGLN	Endoglin isoform 2 precursor	0.64	2	**−2.420**							
gi|46909600	ADA15	A disintegrin and metalloproteinase domain 15 isoform 6 preproprotein	0.64	3	**−2.370**							
gi|8393009	FFR	Chromosome 11 open reading frame2	0.64	2	**−2.346**							
gi|109255232	CE170	Centrosomal protein 170 kDa isoform gamma	0.65	2	**−2.330**							
gi|63176611	SLTM	SAFB-like transcription modulator isoform a						0.65	5	**−2.051**		
gi|5453958	PPP5	Protein phosphatase 5, catalytic subunit	0.66	3	**−2.249**							
gi|8922331	MGN2	Mago-nashi homolog B						0.66	4	**−1.986**		
gi|30061491	E41L1	Erythrocyte membrane protein band 4.1-like 1 isoform b	0.665	5	**−2.178**							

*^1^Weighted **H:L** ratios, scaled to number of measured peptides in each sample, if detected in both Standard and Proteominer samples*.

*^2^Bolding indicates a significant z-score (95% confidence), either >1.960 or <−1.960*.

*^3^Gene removed from NCBI database*.

**Figure 4:**
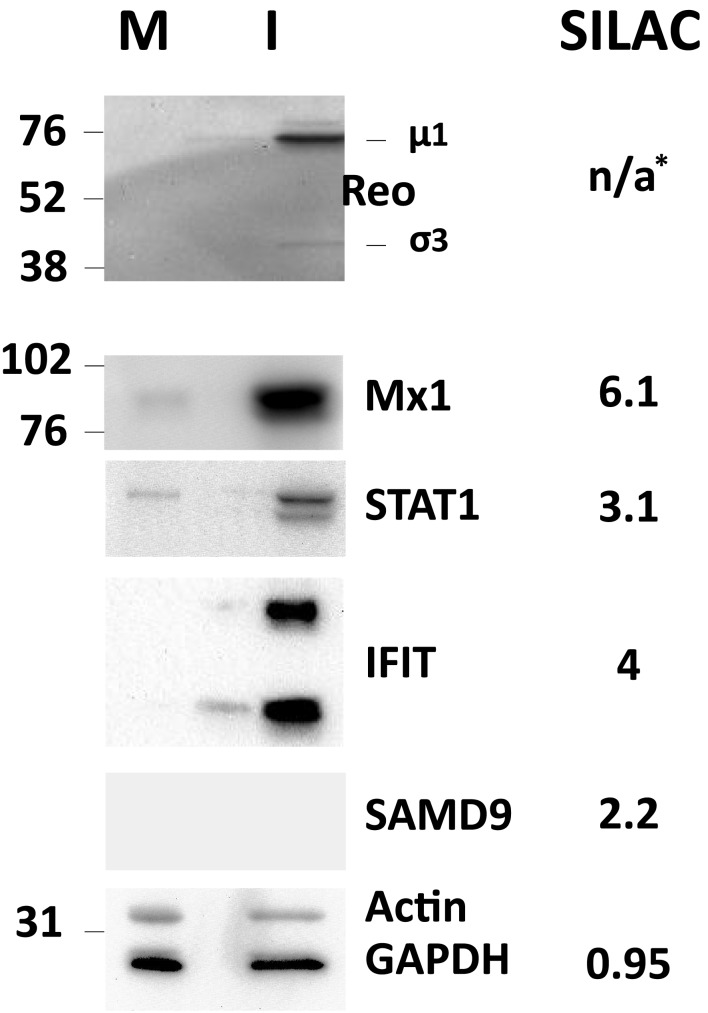
**Western blot validation of experimentally determined SILAC ratos**. HeLa cells were harvested and lysed with 0.5% NP-40 detergent, nuclei removed, and cytosolic fractions dissolved in SDS electrophoresis sample buffer. Proteins were resolved in 10% mini-SDS-PAGE, transferred to PVDF, and probed with indicated antibodies. Bands were visualized, and intensities measured, with an Alpha Innotech FluorChem^®^Q Multi Image^®^ III instrument. Molecular weight standards are indicated at left and SILAC-measured ratios are indicated on the right. *, No viral proteins measured by SILAC as these are absent from mock-infected samples.

Several of the up-regulated and non-regulated proteins that were identified and measured in the SILAC analysis were confirmed by Western blotting (Figure 4). Most Western blot results confirmed the SILAC-determined results although some differences in measured ratios probably reflect different levels of sensitivity of the two assays.

### Proteins up-regulated by reovirus infection are associated with antimicrobial and antiviral responses, GTPase activity, nucleotide binding, interferon signaling, and enzymes associated with energy generation

**Figure 5:**
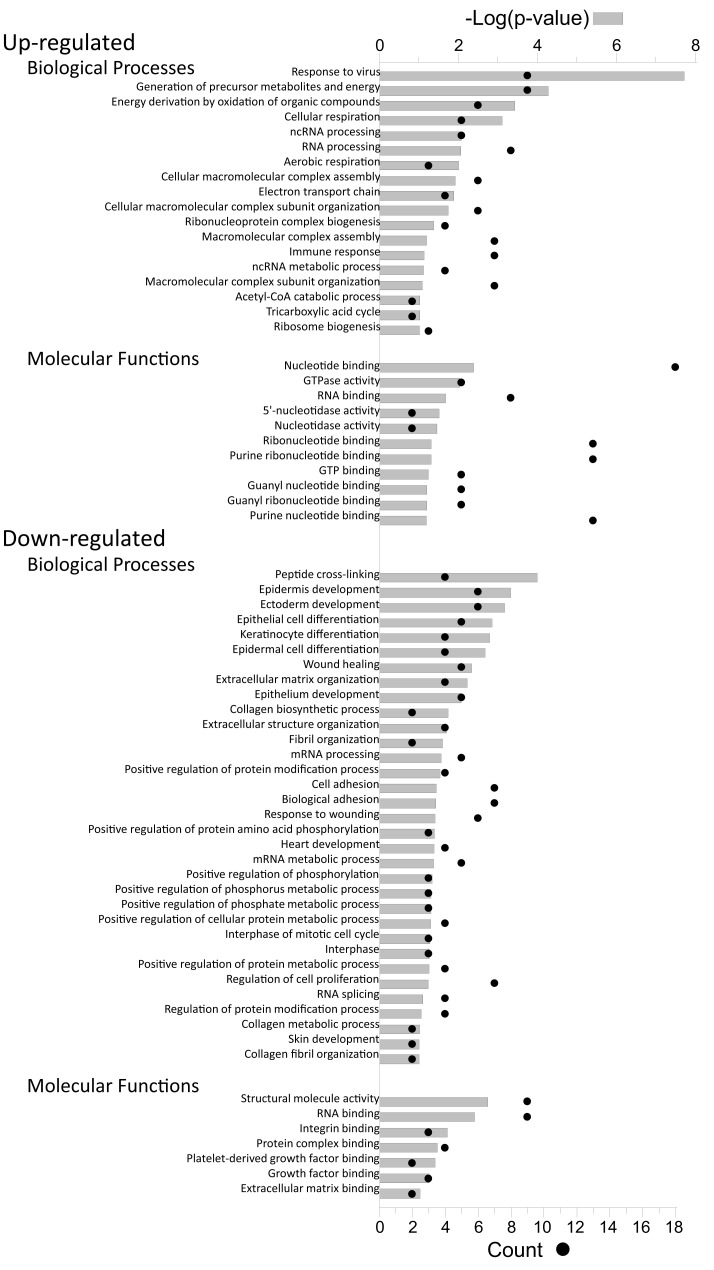
**Gene ontology analyses of up-regulated and down-regulated proteins**. The proteins identified in Table 2 were imported into the DAVID gene ontology suite of programs at the NIAID, gene identifications converted by that program, and ontological functions determined by GOTERM.

Proteins, and their levels of regulation, were analyzed by a variety of means. Protein gi numbers were imported into Uniprot (see text foot note 1) and converted into HUGO nomenclature committee (HGNC) identifiers. The HGNC IDs that represented significantly up-regulated and down-regulated proteins at the 95% confidence interval were then imported into DAVID (Dennis et al., 2003; Huang et al., 2009b), gene identifications converted to Entrez gene IDs by that suite of programs, and gene ontological biological processes and molecular functions identified at 95% confidence (Figure 5).

**Figure 6:**
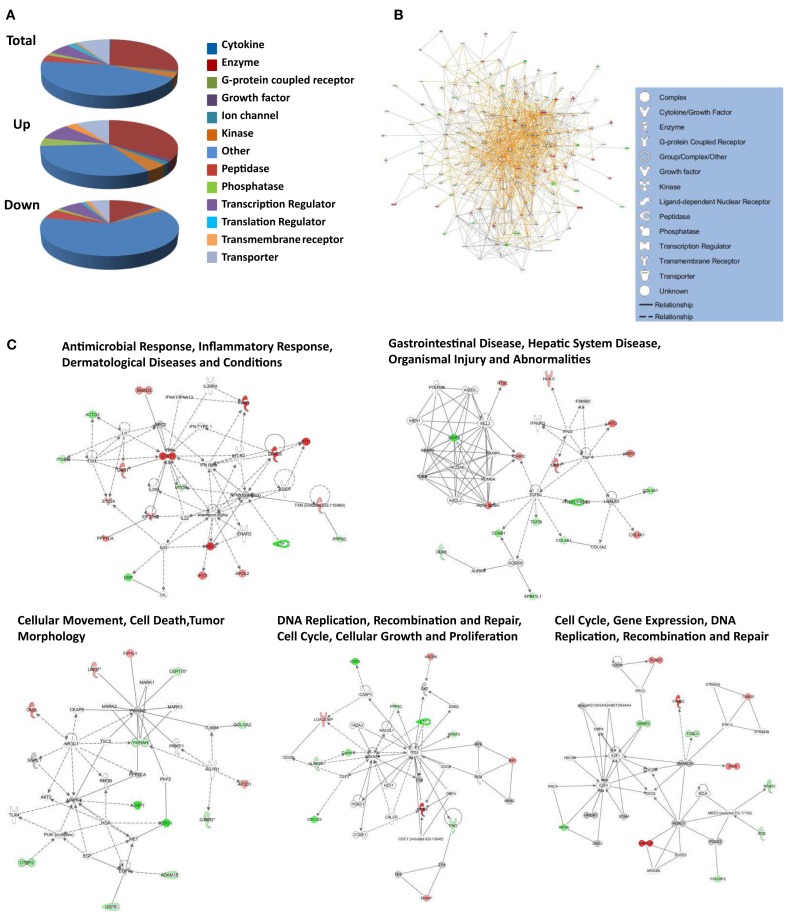
**Molecular pathways of regulated proteins**. Proteins and their levels of regulation were imported into the Ingenuity Pathways Analysis (IPA^®^) tool and interacting pathways were constructed. **(A)** Ontological classifications of all measured proteins (Total) as well as those significantly up- and down-regulated. The indicated ontological classifications start at the top of each pie chart and are presented clockwise. **(B)** Merged networks, containing all molecules present in each of the five individual networks. **(C)** The top five networks, identified at 95% confidence and each of which contained 11 or more “focus” molecules (molecules significantly up- or down-regulated), with pathway names indicated. Solid lines: direct known interactions; dashed lines: suspected or indirect interactions; red: significantly up-regulated proteins; pink: moderately up-regulated proteins; gray: proteins identified but not significantly regulated; light green: moderately down-regulated proteins; dark green: significantly down-regulated proteins; white: proteins known to be in network, but not identified in our study.

Up-regulated proteins were assigned to 18 GOTERM biological processes at 95% confidence (Figure 5, upper), that included cellular respiration, energy metabolism, and responses to viruses. Up-regulated proteins were also assigned to 11 functional groups (Figure 5) including primarily nucleotide binding. Protein gi numbers and levels of regulation were also imported into the Ingenuity Pathways Analysis (IPA^®^) tool which identified 13 GO categories (Figure 6A). Up-regulated proteins were enriched in growth factor, ion channel, kinase, phosphatase, and transmembrane receptor categories, whereas there were proportionally fewer up-regulated peptidase, translation regulators, and “other” (unknown) categories. Interacting pathways were also constructed by IPA. A total of 22 pathways were identified at a confidence level of 95% or greater. Five of these pathways, each with 11 or more “focus” members (significantly up- or down-regulated proteins), shared common members, and it was possible to build a single, merged pathway (Figure 6B). One other pathway (RNA post-transcriptional modification) contained only five focus molecules. The other 16 pathways consisted of several proteins, but contained only a single focus protein (data not shown). The five networks that contained 11 or more focus members corresponded to antimicrobial and inflammatory response; gastrointestinal disease; cell cycle, death, growth, proliferation, and movement; and DNA replication pathways (Figure 6C). Proteins present in the pathways and identified in our analyses as up-regulated are depicted in shades of red and include FADS3, IFIT1, and SAP130. Proteins present in the pathways and identified as down-regulated are shown in green and include AZGP1, LTF, and WDR5. Proteins present in the pathways and identified in our analyses, but neither up- nor down-regulated, are depicted in gray and include NF-KB complex, MAPK1, and TUBB, and proteins known to participate in the pathways but not identified in our analyses are shown in white and include AGER, IL28A, and MARK1–3. IPA analyses identify interaction nodes. For example, several of the highly up-regulated proteins interact with few other proteins, but some, such as STAT, ISG15, and Mx1 interact with four or more. Many of these molecules are involved in innate immunity. In addition, the interferon-induced, large GTPase dynamin-like Mx proteins are important anti viral proteins, particularly against RNA viruses (Haller and Kochs, 2002; Haller et al., 2009) and have been identified in several proteomic studies as up-regulated by influenza virus infection (Baas et al., 2006; Vester et al., 2009; Coombs, 2011). In addition, modulation of interferon response by reoviruses, including through STAT activation, has been demonstrated (Goody et al., 2007; Sherry, 2009; Zurney et al., 2009). Thus, our SILAC observations are validated by, and support, previous findings. Similarly, a few of the down-regulated proteins interact with few partners, but several, including WDR5, appear as interaction “hubs.” We identified numerous other interaction hubs, such as LGAL53 and NF-KB which were not, themselves, significantly altered, but which interacted with several differentially regulated proteins.

### Proteins down-regulated by reovirus infection are associated with cell differentiation, dermal differentiation, and molecular binding

Down-regulated proteins were assigned to 33 biological processes at 95% confidence (Figure 5, lower), that included cell differentiation, peptide cross-linking, and ectoderm and endoderm development. Down-regulated proteins were also assigned to seven functional groups, including structural molecule activity and various factor binding roles (Figure 5). IPA-generated GO categories indicated down-regulated proteins were enriched in unknown categories whereas there were proportionally fewer down-regulated enzymatic and transporter categories (Figure 6A). Additional IPA pathway analyses indicated numerous components of the “Interferon signaling” and “Role of PKR in interferon induction and antiviral response” canonical pathways were significantly up-regulated, whereas numerous arms of the “Regulation of actin-based motility by rho” canonical pathway were down-regulated (data not shown).

### Proteominer enrichment led to identification of comparable numbers of proteins, but PM-enriched proteins were identified by fewer peptides

**Figure 7:**
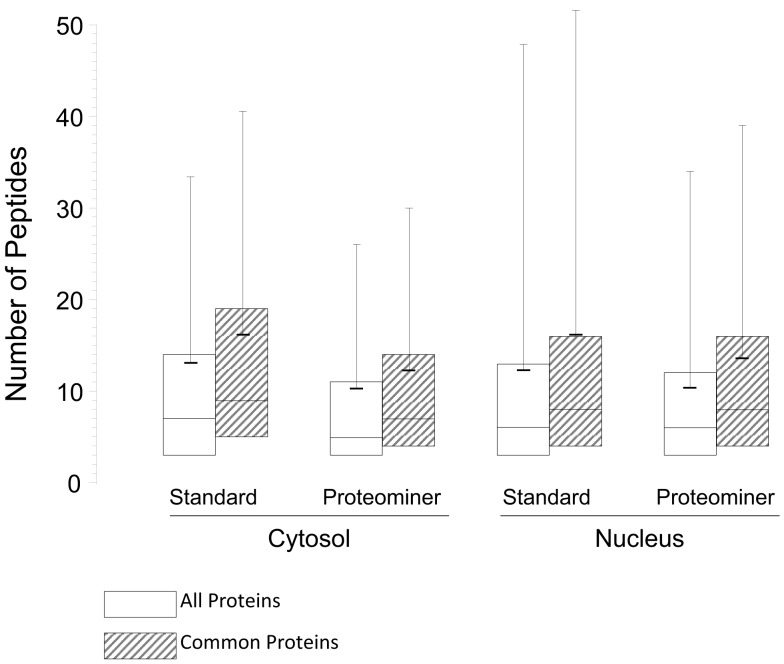
**Box plots of number of peptides identified under each experimental condition**. The box encompasses the upper and lower quartile. Median values for each condition are indicated by the full horizontal line inside each box, the average is indicated by the shorter thick line, and SDs are indicated by upward error bars 
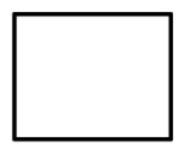
, all identified proteins; 
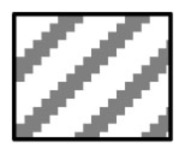
, only proteins identified as common to both the standard and PM analyses.

As indicated earlier, 1,903 proteins were identified in the standard cytosolic fraction, compiled from 24,927 **H:L** peptide pairs (Table 1). This corresponds to an average of 13.1 peptides/protein (SD ± 20.5; Figure 7). In contrast, PM enrichment of the cytosolic fraction led to identification of 17,484 **H:L** peptide pairs and 1,657 proteins (average = 10.3 peptides, ±15.8). Slightly more proteins were identified in the PM-enriched nuclear fraction than in the standard nuclear fraction, but the average numbers of identified peptides, and the corresponding SD, were also lower in the PM-enriched fraction (Figure 7). This pattern was seen irrespective of whether all proteins were examined (white boxes), or only proteins common to both the standard and PM enrichment fractions (gray boxes). Previous studies in our lab have shown that biologic replicates have ∼67% overlap and an *r*^2^ degree of correlation of about 0.04, whereas technical replicates of the same biologic replicate have ∼82% overlap and an *r*^2^ value of about 0.66 (Coombs et al., 2010; Table 3). Comparisons of the overlap and *r*^2^ values between standard preparations and their cognate PM enrichment preparations showed intermediate values of ∼68–74% overlap and *r*^2^ ranging between 0.25 and 0.44 (Table 3), suggesting the PM enrichment strategy did not add substantially to information provided by standard preparations.

**Table 3:** **Correlation and overlap between various sample preparation schemes**.

	Cyto St vs. Cyto PM^1^	Nuc St vs. Nuc PM	Cyto St vs. Nuc St	Cyto PM vs. Nuc PM	Biological replicate^2^	Technical replicate^2^
Percentage of overlap	73.7	67.9	65.4	68.0	67.3	81.5
Overall correlation (*r*^2^)	0.444	0.255	0.159	0.606	0.038–0.057	0.660
Correlation (*r*^2^) for up- and down-regulated proteins only	0.236	0.119	0.046	0.448	0.156–0.174	0.414

*^1^Cyto, cytosolic fraction; Nuc, nuclear fraction; St, standard 2D-LC/MS; PM, Proteominer*.

*^2^Biological and technical values observed in another study Coombs et al. (2010); and unpublished*.

## Note Added in Proof

The Mann laboratory has recently used label-free approaches to determine the relative quantity of each of thousands of proteins in a variety of human cell lines, including HeLa cells (Geiger et al., 2012). As a more direct analysis to determine whether application of Proteominer beads led to identification of lower abundance proteins, we sorted our datasets and determined there were no significant differences in the average and median quantities of proteins identified by either of the two methods in each of the cytosolic and nuclear fractions, further strengthening the main conclusion of this study, that non-biased enrichment using this particular affinity method does not contribute to deeper proteomic mining.

## Author Contributions

Jieyuan Jiang and Kolawole J. Opanubi performed experimental work described herein, all co-authors performed database and computational analyses, and all co-authors wrote and edited the manuscript.

## Conflict of Interest Statement

The authors declare that the research was conducted in the absence of any commercial or financial relationships that could be construed as a potential conflict of interest.

## Footnotes

^1^http://www.uniprot.org/
